# TL1A (TNFSF15) and DR3 (TNFRSF25): A Co-stimulatory System of Cytokines With Diverse Functions in Gut Mucosal Immunity

**DOI:** 10.3389/fimmu.2019.00583

**Published:** 2019-03-27

**Authors:** Vassilis Valatas, George Kolios, Giorgos Bamias

**Affiliations:** ^1^Gastroenterology and Hepatology Research Laboratory, Medical School, University of Crete, Heraklion, Greece; ^2^Laboratory of Pharmacology, Faculty of Medicine, Democritus University of Thrace, Alexandroupolis, Greece; ^3^GI-unit, National & Kapodistrian University of Athens, Third Department of Internal Medicine, Sotiria Hospital, Athens, Greece

**Keywords:** TL1A, DR3, mucosal immunity, inflammatory bowel disease, co-stimulatory

## Abstract

TL1A and its functional receptor DR3 are members of the TNF/TNFR superfamilies of proteins. Binding of APC-derived TL1A to lymphocytic DR3 provides co-stimulatory signals for activated lymphocytes. DR3 signaling affects the proliferative activity of and cytokine production by effector lymphocytes, but also critically influences the development and suppressive function of regulatory T-cells. DR3 was also found to be highly expressed by innate lymphoid cells (ILCS), which respond to stimulation by TL1A. Several recent studies with transgenic and knockout mice as well as neutralizing or agonistic antibodies for these two proteins, have clearly shown that TL1A/DR3 are important mediators of several chronic immunological disorders, including Inflammatory Bowel Disease (IBD). TL1A and DR3 are abundantly localized at inflamed intestinal areas of patients with IBD and mice with experimental ileitis or colitis and actively participate in the immunological pathways that underlie mucosal homeostasis and intestinal inflammation. DR3 signaling has demonstrated a dichotomous role in mucosal immunity. On the one hand, during acute mucosal injury it exerts protective functions by ameliorating the severity of acute inflammatory responses and facilitating tissue repair. On the other hand, it critically participates in the pro-inflammatory pathways that underlie chronic inflammatory responses, such as those that take place in IBD. These effects are mediated through modulation of the relative mucosal abundance and function of Th1, Th2, Th17, Th9, and Treg lymphocytes, but also of all types of ILCs. Recently, an important role was demonstrated for TL1A/DR3 as potential mediators of intestinal fibrosis that is associated with the presence of gut inflammation. These accumulating data have raised the possibility that TL1A/DR3 pathways may represent a valid therapeutic target for chronic immunological diseases. Nevertheless, applicability of such a therapeutic approach will greatly rely on the net result of TL1A/DR3 manipulation on the various cell populations that will be affected by this approach.

## Introduction

Mucosal homeostasis at the gastrointestinal tract requires a delicate co-existence of gut microbiota with the gut-associated mucosal immune system, an interaction that is constantly challenged by environmental factors. Hence, mucosal “health” is depended on an intact genetic structure, preserved by the integrity of the epithelial barrier, and fine-tuned by immunoregulatory responses. Failure of one or more of these balancing elements leads to breakdown of homeostasis and predominance of pro-inflammatory immunological circuits. The latter are critically dependent on key cellular and/or soluble mediators, most prominent among which are cytokines and their receptors. The prototypical disorders that are signified by such dysregulated mucosal immunity are the Inflammatory Bowel Diseases (IBD), in particular Crohn's disease (CD) and ulcerative colitis (UC). Thus, it is of no surprise that cytokines have been the main targets of therapeutic interventions in IBD, which represents a rapidly expanding field in recent years.

TL1A (Tumor necrosis factor-like cytokine 1A) was first reported in 2002 (1). It is a member of the TNF superfamily of proteins (TNFSF) and it is encoded by the *Tnfsf15* gene that is located on chromosome 9q32 in humans and chromosome 4 in mice. TL1A is a type II transmembrane protein with a molecular weight of 28 kDa, which contains 251 amino acids. Similar to other members of the TNF-family, TL1A forms a stable trimer. It exists in a membrane-bound form (mTL1A), which may also be cleaved by matrix metalloproteinases and released as soluble, fully-functional 20-kDa protein (sTL1A) (1, 2).

The functional receptor for TL1A is DR3 (death domain receptor 3), which is encoded by the *Tnfrsf25* gene that is located at the 1p36.3 position in humans (3, 4). DR3 is a type I membrane protein with a 417 AA sequence and a molecular weight of 45 kDa that shares the highest homology to TNFR1 among all members of the TNFRSF. DR3 contains a death domain in its cytoplasmic region; thus it may participate in apoptotic processes. Nevertheless, DR3 signaling also mediates inflammatory/immunological responses. An important characteristic of human DR3 is the existence of several splice variants (13 in humans and 10 in mice). The functional implications of such variety are not fully understood, although encoded proteins may differ in their function (5). To date, the only proven ligand for DR3 is TL1A (including the short variant, TL1/vascular endothelial growth inhibitor).

**Figure 1:**
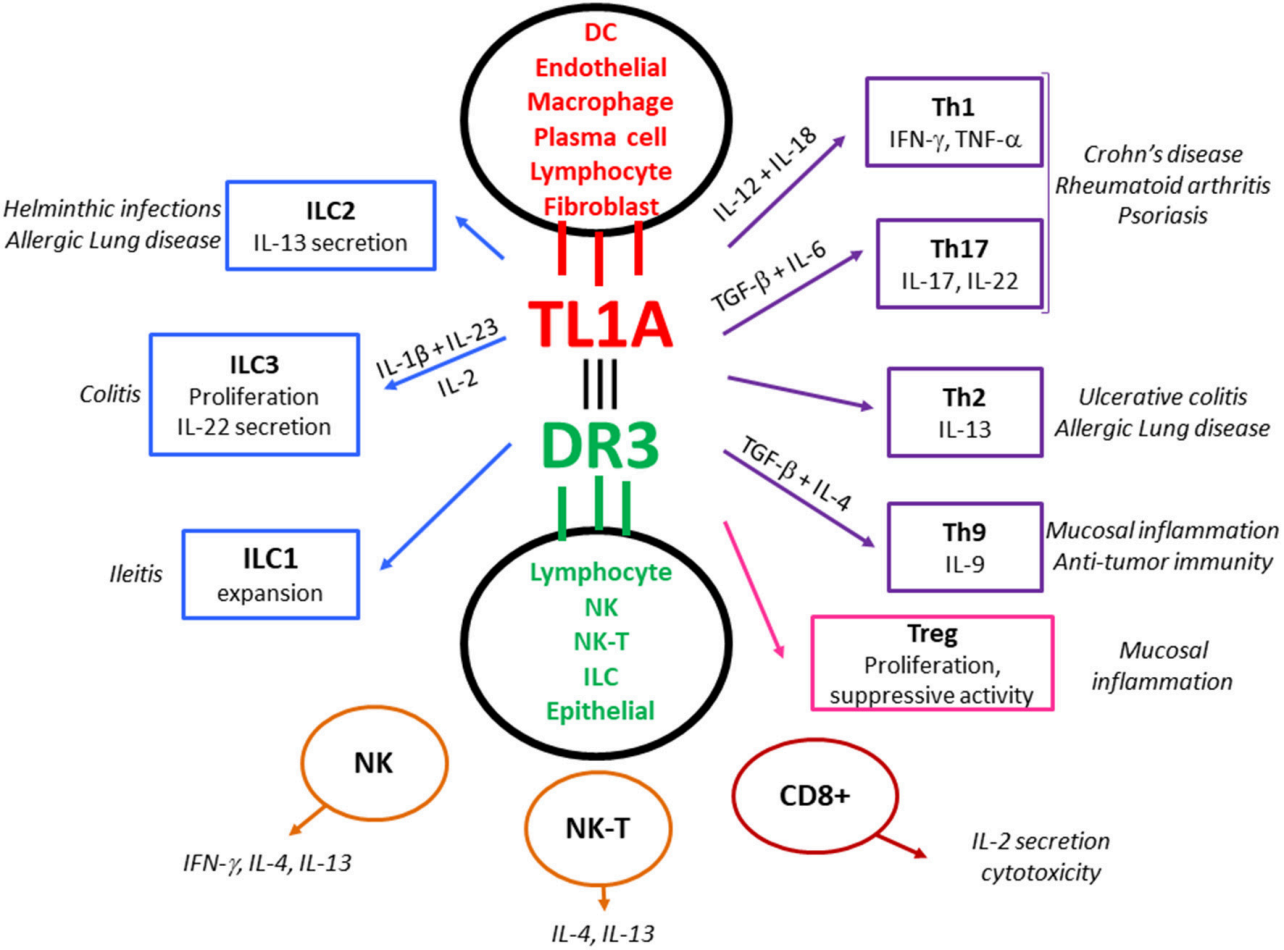
The TL1A/DR3 system as a central regulator of mucosal immune responses, allergy and autoimmunity. TL1A is not constitutively expressed but is induced in mucosal APCs (and other types of immunocytes) following stimulation via microbial and non-microbial antigens. TL1A binds to the functional receptor, DR3, which is expressed by various lymphocytic populations upon activation. TL1A/DR3 signaling enhances proliferation and optimizes cytokine production by responding lymphocytes, acting as a co-stimulatory system that amplifies TCR or cytokine provided signals. This function is of particular importance under conditions of sub-optimal lymphocyte stimulation. All types of effector T cells (Teff: Th1, Th2, Th9, Th17) respond to stimulation with TL1A. DR3 is also expressed by regulatory lymphocytes (Tregs), which proliferate in response to TL1A, although this may be accompanied by a temporary halt of suppressive function, especially in the event of acute inflammation. DR3 expression has also been demonstrated in innate lymphoid cells (ILCs) and DR3 signaling affects their function. Finally, TL1A/DR3 signaling pathways have been reported in NK and NK-T cells, as well as CD8+ lymphocytes. This universal expression of DR3 by innate and adaptive effector and regulatory populations implies a key regulatory role of the TL1A/DR3 system in mucosal immunity. Alongside, experimental data from animal models and translational data from patients indicate an important contribution of the TL1A/DR3 system in allergic lung inflammation and autoimmune diseases such as Crohn's disease, Ulcerative colitis, Rheumatoid arthritis, and Psoriasis.

There is now able evidence that interactions between TL1A and its functional receptor DR3 affect gut mucosal immunity both during homeostatic conditions and in various inflammatory states (Figure 1). In particular, their role in IBD is supported by a variety of genetic, immunological, experimental, and translational data. The current review aims to critically present existing literature on the role of TL1A and DR3 in mucosal immunity.

## *Tnfsf15* Polymorphisms Affect Susceptibility to Intestinal Diseases

A first line of evidence for the potential importance of TL1A in the pathogenesis of IBD is derived from studies that reported significant associations between genetic variations in the *Tnfsf15* gene and susceptibility to IBD (Table 1).

**Table 1:** Genetic associations of *Tnfsf15* gene with susceptibility and phenotype of IBD.

**Polymorphism**	**Ethnicity**	**Susceptibility/Phenotype**	**References**
rs3810936	Asian, Caucasian Asian	CD UC	(6)
rs6478108	Asian, Caucasian Asian, Caucasian Asian	CD UC CD B2/B3	(6) (7) (8) (9)
rs4979462	Asian	CD	(6)
rs6478109	Asian, Caucasian Asian	CD UC	(6) (7) (10) (6)
rs7848647	Asian, Caucasian Asian Caucasian, Asian	CD UC CD early onset	(6) (7, 11)
rs7869487	Asian, Caucasian	CD	(6)
rs4263839	Caucasian Caucasian Asian	Colonic CD location Bowel resection CD, B2/B3	(12) (13) (14)
rs4574921	Asian	CD B3p	(9)
rs11554257	Caucasian	MR-UC	(15)
rs3810936	Asian Asian Asian	Severe CD CD CD B3p	(16) (17) (7)
rs4246905	Asian	IBD	(18)

*MR, Medically refractory; B2, B3, p, Montreal classification indicators for Crohn's disease*.

In 2005, Yamazaki et al. were the first to report that a specific genetic variant of *Tnfsf15*, tnfsf15_28, was strongly associated with susceptibility to IBD in Japanese patients, whereas the gene was monomorphic in a Caucasian population from the UK (19). Further investigation of these two ethnic groups revealed 5 different SNPs, tnfsf15_26, 31, 35, 36, and 41, that were polymorphic in both groups, forming three different haplotypes which affected susceptibility to IBD. In both ethnic groups, haplotype A was identified as a high-risk marker for susceptibility to IBD, whereas haplotype B was found to be a low-risk genetic factor. Haplotype C was not significantly associated with IBD risk in either population, despite its frequent detection. Two years later, Picornell et al. investigated the aforementioned three haplotypes in Jewish and non-Jewish IBD and control populations in Los Angeles, USA. In the non-Jewish population, similar to the previous study, haplotype B was less frequent in both CD and UC patients compared to controls, highlighting a possible protective role (20). On the other hand, no association of haplotype A with IBD was seen in either population, suggesting that *Tnfsf15* polymorphisms are ethnic-specific. These findings were further supported by independent studies from Asia and Europe.

Interestingly, haplotype B of the *Tnfsf15* gene has been found to affect protein production by monocytes and macrophages. Peripheral CD14+ monocytes and monocyte-derived macrophages from patients carrying Haplotype B or the rs6478109 A minor allele produce higher levels of TL1A in response to FcγR or LPS stimulation (21, 22). Interestingly, haplotype B has been reported to confer CD risk in Jewish patients but the rs6478109 A minor allele conferred protection to subjects of European descent. However, the studies on the effect of *TL1A* gene Haplotypes on membrane bound TL1A has provided conflicting results. Earlier studies have shown that membrane expression of TL1A was up-regulated on peripheral monocytes from Jewish but not from non-Jewish CD patients that carry the B haplotype but later studies have shown increase membrane bound TL1A in homozygotes of the protective rs6478109 A allele in European descent subject (21, 22).

A Korean study in pediatric CD patients and adult controls showed that six specific haplotypes of the *Tnfsf15* gene were more frequently reported and, of those, two were significantly different between the two groups; the haplotype including T-C-A-T-C SNPs (rs3810936-rs6478108-rs6478109-rs7848647-rs7865494) was more frequent in controls, whereas haplotype including C-T-G-C-C SNPs in CD patients (7). On the other hand, in a Chinese study consisting of 13 pediatric patients with Very-early-onset IBD (VEO-IBD), no association between *Tnfsf15* gene mutations and VEO-IBD was found and this finding may be the result of either ethnic differences or the small number of patients included in the study (23). Contrary to the previous study, it was recently shown that the *Tnfsf15* rs4246905 SNP was associated with development of CD in children with chronic granulomatous disease (24).

The importance of the ethnic background for *Tnfsf15* polymorphisms was further highlighted in an Indian study showing that haplotype A was significantly more frequent in IBD cases than in healthy individuals, while the opposite was observed for haplotype C. Further investigation identified two additional SNPs (rs10114470 and rs4263839) and generated 7 different haplotypes, from which haplotype H had a possible protective role being more frequent in healthy individuals, whereas haplotypes I and J conferred susceptibility to IBD (25). Haplotypes of the *Tnfsf15* gene that are associated with either susceptibility or protection from CD have also been reported in the European population (26). Similar to Yamazaki, a Korean study showed that the allele T of the SNP rs6478108 is indeed a “risk” allele as it is more frequently found in patients with UC (8). In a recent study that included a large number of IBD patients and healthy individuals across Europe and Asia showed that, although allele frequencies are similar between the European and Asian population, *Tnfsf15* variants have a stronger association with IBD susceptibility in people originating from Asia (18).

Other studies have also proposed that certain *Tnfsf15* alleles may bear prognostic value for the severity of IBD. In the Chinese population, the T allele of SNP rs10114470 was associated with increased probability of developing stricturing, penetrating, or perianal complications (27). In the same notion, Pernat Dobrez et al. identified the SNP rs4263839 as a possible marker for disease progression. In their study, 72.2% of CD patients who were carriers of allele A had progressed from an inflammatory (B1) to stricturing (B2) or penetrating (B3) phenotype, as compared to only 55% of those bearing the allele G (28). In another study, polymorphisms in the *Tnfsf15* gene were associated with medically refractory UC (15). The susceptibility loci for CD reported by Yamazaki et al. were also detected in CD patients from Ryukyu Islands, near Japan. Interestingly, CD patients, bearing the risk alleles, had increased bacterial abundances of *Bacteroidetes* and more specifically, of *Prevotella*, but whether the genetic background is the outcome of microbiome composition alteration or *vice versa*, remains unclear (29).

Interestingly, besides IBD, *Tnfsf15* genetic variants have also been associated with other GI diseases, such as diverticulitis and Irritable Bowel Syndrome (IBS). Connelly et al. discovered that the SNP rs7848647 is highly associated with diverticulitis and that haplotype carriage may predict the need for surgical intervention (30). Additional protective or risk-conferring haplotypes for diverticulitis were reported later by the same group (31) Regarding IBS, Zucchelli et al. reported a strong association between the SNP rs4263839 and patients suffering from IBS. In particular, the G allele of the SNP rs4263839 was identified as a high-risk marker and further investigation revealed that it could lead to higher expression levels of TNFSF15 in healthy individuals (32).

Taken together, various SNPs of the *Tnfsf15* gene have been associated with not only IBD, but also diverticulitis and IBS, and seem to be promising predictors for intestinal disease susceptibility and/or progression. Nonetheless, the interpretation of the role of *Tnfsf15* mutations seem to be influenced by ethnic background, as a stronger association with Asians has been reported. In addition, no definitive functional implications for existing, risk-associated polymorphisms have been reported. Nevertheless, a few studies have proposed that certain polymorphisms may be associated with specific functional effects, a finding that, interestingly, was also affected by the ethnic background in some cases (22, 33, 34).

## The Complex Role of tl1a/dr3 in Mucosal Immunity

### Expression, Regulation, and Function of the TL1A/DR3 System in Immune Cells

#### Mononuclear Phagocytes

The cellular sources of TL1A and requirements for its expression indicate its important role in innate and adaptive immune responses especially at the intestinal mucosa. Besides the original description of its constitutive expression in endothelial cells (1), TL1A was also found to be expressed by mononuclear phagocytes at the intestinal lamina propria of both mice and humans under inflammatory conditions. In particular, early studies in murine models of ileitis and colitis have shown that TL1A was mainly expressed by CD11c^high^/MHC-II^+^ mononuclear phagocytes of the lamina propria and the MLNs and CD11c^low^/MHC-II^−^ mononuclear phagocytes of the lamina propria that probably represent dendritic cells and macrophage subsets (35, 36). In patients with CD or UC immunolocalization of TL1A was reported in tissue macrophages and lymphocytes, as well as in infiltrating plasma cells in UC (37). A recent study has further characterized lamina propria mononuclear phagocytes that primarily produce TL1A in mice, CD patients and healthy subjects and reported expression of CD14 and CX3CR1 surface markers, which classifies them as antigen sampling mucosal macrophages. TL1A production by CD11c^+^CX3CR1^+^ mononuclear phagocytes correlated with disease activity in CD in humans. In mice CD11c^+^CX3CR1^+^ mononuclear phagocytes produced TL1A, in a MyD88-dependent fashion, in response to mucosal-adherent bacteria (38).

The abundant cellular expression of TL1A in APCs, led to studies on the regulation of its expression in this particular population. These studies showed that TL1A expression by APCs is responsive to signaling through FcRγ receptors, TLRs or microbial antigens. In particular, in monocytes and monocyte-derived dendritic cells (DCs) TL1A mRNA and protein (both soluble and transmembrane forms) was highly induced by FcRγ stimulation through plate-bound, cross-linked human IgG (39). A potential clinical relevance of this association was implied in two studies in rheumatoid arthritis (RA). Cassatella et al. reported that mononuclear phagocytes strongly expressed TL1A in rheumatoid factor–positive but not rheumatoid factor–negative patients (40). *In vitro* stimulation of monocytes with various preparations of insoluble immune complexes led to significant upregulation of TL1A (40). Furthermore, Bamias et al., demonstrated that soluble TL1A concentrations were significantly higher in rheumatoid factor–positive than rheumatoid factor–negative patients (41). In addition to stimulation through the FcRγ receptor, bacterial signals also upregulate the expression of TL1A. In the monocytic cell line U937, TL1A was induced by LPS. This pathway involved activation and binding of the transcription factor nuclear factor kappa-light-chain-enhancer of activated B cells (NFkB) to a specific binding site in the 5′ flanking region of TL1A (42). LPS responsiveness was also confirmed in bone-marrow derived dendritic cells (43).

Shih et al. studied the effect of stimulation by cultured microorganisms on TL1A expression by monocytes or DCs (44). Cells were stimulated with a variety of cultured microorganisms (gram-negative [*Escherichia coli, E. coli* Nissle 1917, and *Salmonella typhimurium*], gram-positive [*Listeria monocytogenes* and *Staphylococcus epidermidis*], partial anaerobes [*Campylobacter jejuni*], and obligate anaerobes [*Bacteroides thetaiotaomicron, Bifidobacterium breve*, and *Clostridium* A4]). In all cases, upregulation of TL1A was seen through an NF-kB (as well as p38 MAPK)–dependent mechanism. TLR signaling could only partially substitute for the effect of whole bacteria stimulation. Moreover, TLR7/8 signaling demonstrated a negative effect by significantly decreasing IC-induced TL1A expression of monocytes (45). In another study, Kamada et al. also reported TL1A induction by heat-killed bacteria in CD-associated LP macrophages (46).

#### Effector Lymphocytes

TL1A is also expressed on activated lymphocytes, indicating the formation of positive regulatory loops during mucosal inflammation (47, 48). Especially in the small bowel, membrane bound TL1A is expressed by activated gut specific CCR9^+^ lymphocytes (47) and it is possibly up-regulated by stimulation through the T-cell receptor (TCR) (i.e., by phytohemagglutinin) (49).

Expression of DR3, the cognate receptor of TL1A, is mainly detected in lymphocytic populations, mostly following activation. DR3 signaling enhances CD4+ lymphocyte proliferation by increasing both IL-2 production and expression of IL-2RA and IL-2RB (1). The effect of TL1A co-stimulation is particularly important in conditions of sub-optimal stimulation via CD3 or CD28 (43). TL1A preferentially enhances proliferation of murine memory CD4+ T cells (35), but can also induce mild proliferation and strong IL-2 and IFN-γ expression by naïve T cells (43). TL1A co-stimulation of CD4+ T cells results in production of multiple cytokines including IL-2, IL-4, IL-13, interferon-gamma (IFNγ), and IL-17 (1, 43, 47, 48, 50). Although, TL1A-DR3 interaction enhances T cell proliferation and is required for optimal cytokine production, it appears to be dispensable for differentiation of naive T cells into Th1, Th2, or Th17 effector cell subtypes (43).

TL1A-DR3 is proposed as an important system for enhancement of immune responses in the periphery. Indeed, TL1A acts in synergy with IL-12 and IL-18 to enhance adaptive Th1 and innate IFNγ responses by T cells (49, 51, 52). Specific T cell subsets that up-regulate IFNγ and TNFα production in response to TL1A and IL-12/18 co-stimulation have been characterized by the expression of CCR9 (47), CD161 (53), and IL-18Rα (52). Interestingly, TL1A responsive T cells were preferentially localized at the intestinal mucosa, which indicates a prominent role of TL1A in intestinal IFNγ-mediated immune responses. Consistent with its role as a non-specific co-stimulator, irrespective of T-cell lineage commitment, TL1A was shown to enhance secretion of Th2 cytokines by activated T cells and amplified IL-13 production by NKT cells in a murine model of allergic lung inflammation (43, 48). The role of TL1A in Th17 responses appears more complex. Pappu et al. found that TL1A^−/−^ dendritic cells exhibited reduced ability to support differentiation of Th17 lymphocytes and TL1A co-stimulation was required for optimal antigen-independent proliferation of differentiated Th17 lymphocytes (50). In contrast, Jones et al. reported that TL1A-DR3 interactions inhibit polarization toward the Th17 lineage but support IL-17 production in fully committed Th17 cells (54). Differences of *in vitro* experimental conditions for Th17 polarization may account for the aforementioned discrepancies. Furthermore, TL1A has been found to induce IL-22 production by human peripheral memory CD4^+^ T cells and committed Th17 cells, through up-regulation of IL-9 (55). Nevertheless, TL1A co-stimulation has been proven essential for both gut and cerebral immunopathology that depends on Th1 and Th17 in relevant mouse models (36, 43, 50).

#### Tregs

Control of proliferation and suppressive function of regulatory T cell (Treg) is another way through which the TL1A-DR3 system controls local immune responses. Tregs constitutively express DR3, and DR3 signaling has been found to enhance proliferation of Tregs partly by enhancing their responsiveness to IL-2 (56–58). The proliferative effect of TL1A on Tregs has been recently confirmed for human cells in *ex vivo* studies (59). However, TL1A inhibits Treg suppressive ability both directly and indirectly by rendering activated Teff cells resistant to Treg mediated suppression (56, 57). Removal of TL1A completely restores the suppressive ability of Tregs both *in vitro* and *in vivo* (56, 58). Given the transient nature of TL1A up-regulation by APCs, an accelerated effector T cell response could be accompanied by increased numbers of Tregs capable of controlling activated T cells when local concentrations of TL1A have decreased. These findings suggest the existence of an elegant operational system that promptly amplifies immune responses against invading pathogens and rigorously dampens immune activation once the pathogens is eliminated (60). Finally, the addition of TL1A in conventional T cells cultured under FoxP3-promoting conditions, inhibited iTreg differentiation (57, 61). Instead, forced overexpression of high levels TL1A by FoxP3-expressing T cells promoted proinflammatory characteristics such as the production of IL-4 and IL-13 (61). TL1A-overexpressing Tregs were unable to protect from colitis in the T cell transfer colitis model (61). However, transgenic Tregs expressing low levels of TL1A were able to suppress T cell transfer colitis, an effect dependent on DR3 signaling and associated with protective levels of IL-17 and TGFβ (61). The importance of this low level expression of TL1A for the maintenance of Treg populations and functions in the periphery remains to be elucidated.

#### Th9 Cells

Although the TL1A-DR3 system had no effect on Th1/Th2 polarization and a debated effect on Th17 differentiation, it recently became evident that it plays an important role for the generation of Th9 cells that are involved in defense against helminthes and allergy. On the one hand, TL1A co-stimulation enhances generation of Th9 T cells in the presence of TGFβ and IL-4, conditions that favor Th9 differentiation (62). On the other hand, in the presence of TGFβ and IL-2, conditions that favor iTreg generation, TL1A diverts the differentiation of iTregs to Th9 cells (62). Interestingly, TL1A up-regulates IL-9 secretion though an alternative pathway that involves STAT5 activation by IL-2 instead of STAT6 activation by IL-4 (62). *In vitro* data have also been coupled by *in vivo* evidence of an increased pathogenicity of Th9 cells in the presence of TL1A in a model of Th9-dependent allergic ocular and lung inflammation (62). In addition to T cell dependent allergic lung inflammation, intact DR3 signaling has also been found important for ILC2 expansion and pathogenicity in innate models of allergic lung inflammation (63). Besides allergic immunopathology, TL1A-directed Th9 polarization, has been associated with CD4^+^-dependent anti-tumor responses. Dectin-1-activated dendritic cells, acting partly though TL1A-DR3, have been found to induce Th9 cells that enhance tumor-specific CTL activity against OVA-expressing melanoma tumors (64).

#### Other Cell Populations

TL1A co-stimulation enhances proliferation, IL-2 production, and cytotoxicity of DR3 expressing CD8+ T cells (65). Moreover, natural killer (NK) cells are capable of expressing DR3 after stimulation with IL-12 and IL-18, which led to enhanced IFNγ production and anti-tumor responses following TL1A stimulation (51, 66). Innate lymphoid cells (ILCs) also express DR3 and increase cytokine production upon TL1A stimulation (63). These findings indicate a broader role of TL1A/DR3 system in protective immunity. DR3 is also highly expressed on NKT cells. In this population, unlike in T cells, TL1A appears to promote a more restricted set of cytokines, enhancing IL-4 and IL-13 but not IFNγ production (48). B cells also express DR3, especially after polyclonal stimulation through the B-cell receptor (67). Plasma cells (but not B cells) also expressed very high levels of DR3 in a mouse model of collagen-induced arthritis (68). However, the role of TL1A/DR3 in B cell functions is not yet clear.

### Functional Roles of TL1A/DR3 in Mucosal Homeostasis and Inflammation

The original identification of TL1A in 2002 was followed by an abundance of studies that have largely brought about the significance of the TL1A/DR3 system in immunological responses, with particular emphasis in mucosal immunity pathways. Although originally presented as Th1 polarizing molecules, TL1A and DR3 were soon proved to display a vast array of multiple and even opposite immune functions that are critically dependent on the particular clinical or experimental scenario. In this process the contribution of genetically manipulated murine models and the application of neutralizing or stimulatory monoclonal antibodies have been of paramount importance (Table 2).

**Table 2:** Effects of genetic or immunological manipulation of TL1A/DR3 expression.

**Model**	**TL1A Tx**	**TL1A-Tg**	**TL1A ko**	**DR3 ko**	**Anti-TL1A**	**References**
Spontaneous phenotype		Ileitis, TH2				(56, 57, 69)
DSS			Worsening	Worsening	⇓ Chronic	(36, 70)
TNBS					⇓ Weight loss ⇓ Histology	(57)
SAMP ileitis				Protected		
Gai2 ko transfer					⇓ Weight loss ⇓ Histology	(36)
Experimental allergic encephalomyelitis (EAE)			⇓ Clinical score	⇓ Clinical score		(43, 50)
Collagen-induced arthritis (CIA)	Worsening				Protected	(71)
Antigen-induced arthritis (AIA)	Worsening			⇓ Chronic arthritis	Protected	(71)
Ovalbumin (Ova) lung hypersensitivity pneumonitis				Protected	Protected	(43, 62)

#### Protective Functions During Acute Injury and Repair: The Role of TL1A/DR3 in Innate Immunity

Recent studies have provided evidence for a protective role of TL1A/DR3 in host defense against acute harmful stimuli. Buchan et al. demonstrated expression of TL1A by F4/80^+^ macrophages in the spleen of mice during *Salmonella enterica* Typhimurium infection (72). In the same model, DR3 signaling was essential for optimal expansion of activated/memory CD4^+^ T cells that produced IFNγ and facilitated bacterial clearance (70, 72). Similarly, in the absence of DR3 signaling, early antiviral immunity against murine cytomegalovirus was impaired and fewer virus-specific CD4+ and CD8+ cells were generated, resulting in increased viral loads (73). A recent study by Pham et al. further showed that DR3 signaling is essential for non-cognate stimulation of Th1 cells and effective elimination of intracellular bacteria in mice (74). Studies on human memory T cells have also shown that TL1A and IL-15 synergize to enhance proinflammatory cytokine production independently of cognate TCR–MHC-II interactions (52). They describe a significant population of memory CD4^+^ T cells characterized by the expression of IL-18R and DR3 and located preferentially in mucosal surfaces such as the small intestine, the colon, the nasal mucosa and the skin. Stimulation with TL1A/IL-15 induced strong IFNγ responses accompanied by production of IL-6, TNF-α, GM-CSF, IL-5, IL-13, and IL-22 with concomitant suppression of IL-10 production. These findings imply that TL1A/DR3 supports the innate activity of mucosal memory T cells. Whether these cells exert protective mucosal roles during acute inflammation, however, remains to be shown. On the other hand, identification of large numbers of these IL-18Rα^+^DR3^+^ T cells has been reported in the small bowel of CD patients. This rather indicates a primary pathogenetic/proinflammatory role for TL1A in chronic autoimmune intestinal inflammation (74).

Further support for an important role of TL1A/DR3 signaling in mucosal homeostasis arose from the recent discovery of the stimulatory effect of TL1A on innate lymphoid cells (ILCs). Type 2 cells (ILC2) at mucosal surfaces may depend on TL1A/DR3 signaling, as high DR3 expression was detected in both human and murine ILC2. Stimulation with TL1A resulted in enhanced expansion, survival, and function of ILC2 (75). More importantly, this effect was independent of the critical ILC2 regulators IL-25 and IL-33. The biological significance of these experimental findings were substantiated by the increased susceptibility of DR3^−/−^ lymphopenic mice to gut helminthic infections (75) and their failure to develop lung responses to nasal challenge with papain (75). Finally, Meylan et al. reported that TL1A-dependent co-stimulation of ILC2 was involved in experimental allergic lung disease (63).

Group 3 ILCs are defined by their expression of RORγt^+^ and ability to produce IL-17 and IL-22 (76). They reside mostly at the intestinal mucosa to enhance intestinal barrier integrity and epithelial repair primarily through the production of their signature cytokine IL-22 (77). ILC3-derived IL-22 is critical for constraining commensal bacteria and protecting against pathogenic bacteria and viruses via the regulation of intestinal anti-microbial peptides and B cell responses (78–84). TL1A is one of the intermediate messengers of innate immune responses produced by ILC3s that rescue mice from *C. rodentium*–induced colitis. Specifically, bacterial sensing CX3CR1^+^ mononuclear phagocytes in the intestinal lamina propria produce TL1A, IL-1β, and IL-23 that upregulate production of IL-22 from intestinal ILC3s and protect against infectious colitis and dextran sodium sulfate (DSS)-mediated acute colitis (38, 85). Up-regulation of IL-22 production was depended on DR3 signaling in both mouse and human ILC3 that constitutively express this receptor (85, 86). Furthermore, the combination of IL-1β, IL-23 and TL1A, induced the expression of CD25 on human ILC3 cells to enhance IL-2-mediated proliferation (86). Collectively, the above findings indicate that TL1A/DR3 have a major role in orchestrating innate immunity pathways in the intestine by the regulation of the local ILC3 pool and cytokine production.

The role of TL1A/DR3 in preserving mucosal homeostasis was shown with the use of TL1A- and DR3-deficient mice (70). DSS-colitis was more severe in the absence of either TL1A or DR3, indicating protective roles for these proteins during acute mucosal injury and repair. This was associated with a compromised ability to maintain adequate numbers of Foxp3^+^ regulatory T cells in the periphery and inability to restrain Th17 immune responses. Similar results have been observed in the T cell transfer model, where intact DR3 signaling and a low level of TL1A expression on Foxp3^+^ regulatory T cells was required to maintain their suppressive function and rescue from colitis (61). It should be noted, however, that in the DSS model the effects of TL1A and DR3 deficiency were not identical as there were differences between the two cases in regards to mortality and kinetics of inflammatory responses (70). However, Castellanos et al have failed to detect significant differences in Foxp3^+^ T regulatory or IL-17-producing RORγt^+^ (Th17) cells during acute colitis in DR3-deficient mice (38). Using mice with ILC3 specific DR3 deletion (DR3^ΔILC3^) they showed that exacerbation of DSS colitis was due to the downregulation of IL-22 production by ILC3s, whereas treatment with recombinant IL-22 rescued survival of DR3^ΔILC3^ mice (38).

#### Pro-inflammatory Functions During Chronic Inflammation: The Role of TL1A/DR3 in Adaptive Immunity

Many studies during the last decade have demonstrated that the TL1A/DR3 system is up-regulated in patients with IBD and chronic intestinal inflammation. Gut tissue specimens from CD and UC patients exhibit increased TL1A transcripts and protein expression, which correlated with the severity of tissue inflammation. Paired samples of macroscopically uninvolved intestine from the same patients had intermediate levels of TL1A, and minimal or un-detectable amounts were observed in healthy controls (37, 49). Recent studies have correlated the increase of TL1A transcripts at the colonic mucosa of IBD patients to the levels of IL-17A expression (87). Immunohistochemical studies and cytometric analysis on isolated cells from the lamina propria further specified that the increased amounts of TL1A detected in IBD tissue were derived from infiltrating lymphocytes and intestinal macrophages in CD and plasma cells in UC. A similar pattern of expression, which correlated with disease activity, was observed for DR3 (37, 49). Furthermore, systemic levels of TL1A and its decoy receptor DcR3 parallel disease activity in colonic CD and UC. Recently, a significant correlation of the expression of DR3 on peripheral blood mononuclear cells with CRP levels was observed in newly diagnosed children and adults with IBD (88). Both local expression and systemic levels were found to decrease following effective treatment (88–90).

Studies in animal models with transgenic expression of TL1A provided more mechanistic insights about the possible involvement of TL1A in IBD. Mice with forced constitutive expression of TNFSF15 (TL1A-tg) in either the lymphoid or myeloid cell compartments demonstrated a stable phenotype of mild ileitis (56, 57, 69). All transgenic mice developed inflammatory changes in the terminal ileum that included disrupted villi architecture, infiltration of the lamina propria with inflammatory cells, goblet cell hyperplasia and thickening of the muscularis propria. Inflammatory changes were accompanied by lengthening of the small intestine and failure to gain weight. The patchy distribution of the inflammatory lesions and the development of intestinal fibrosis are two characteristics shared with human CD. Small bowel pathology was associated with increase in activated T cells and regulatory Foxp3^+^CD4^+^ T cells. The most prominent feature was a predominant Th2 mucosal response. Indeed, TL1A-Tg mice displayed elevations in mucosal IL-13 and IL-5 mRNA content, whereas, blockade of IL-13 ameliorated the severity of ileitis (56, 57, 69). The striking resemblance of the TL1A–induced pathology to intestinal anti-parasitic responses led the investigators to further study the role of TL1A in this field. They subsequently observed that TL1A can stimulate IL-13 production by Group 2 Innate lymphoid cells, an effect mediated via DR3 (63). However, the TL1A-dependent innate pathway they identified was not required for effective intestinal anti-parasitic responses but mostly played a major role in allergic lung pathology in relevant murine models (63).

It is now widely accepted that Th2 predominant immunity is a characteristic of the late maintenance stages of clinical and experimental IBD, as it was shown in great detail in CD-like ileitis in SAMP1/YitFc mice (91–94). Interestingly, both TL1A-Tg and SAMP1/YitFc mice develop small intestinal inflammation, marked hypertrophy of the muscular layer, and high mucosal expression of IL-13 and IL-5. Additionally, mucosal TL1A mRNA expression is also upregulated in the chronic phase of SAMP1/YitFc ileitis (35). These studies raise the possibility that the proinflammatory function of TL1A may be partially mediated through induction of Th2/IL-13 dependent mucosal responses, which are now recognized as central pathogenetic factors in IBD. The importance of TL1A/DR3 in murine colitis has also been investigated in the TNBS, DSS and the G-protein ai2 deficient models (36, 57). Development of colitis was associated with mucosal upregulation of TL1A and DR3 and colitis was effectively prevented or attenuated by the administration of anti-TL1A neutralizing antibodies. Taken together, mucosal overexpression of TL1A (primary or secondary) may be implicated in the induction of pathogenetic effector proinflammatory pathways.

A functional dichotomy between membrane and soluble forms of TL1A may also occur, as it was shown recently. Using a membrane restricted TL1A transgenic mouse Ferdinand et al., have shown that increased production of soluble TL1A, possibly by APCs, was required to produce maximal small bowel pathology. In contrast membrane bound TL1A, mostly of T-cell localization, was required to elicit inflammatory responses including IL-13, IL-17, and IL-9 production in murine lungs (95).

DR3 and TL1Ako mice do not develop any gross abnormalities. Nevertheless, immunological characterization of these strains detected specific defects that may be of interest. It was shown that DCs from TL1A^−/−^ mice fail to support the differentiation and proliferation of Th17 lymphocytes (50). Consistently, these mice were protected from Th17-mediated inflammation in a model of Experimental Autoimmune Encephalomyelitis (43, 50). Similarly DR3 signaling was required for Th2-mediated lung immunopathology in an Ova model of allergic lung inflammation (43). TL1A deficiency has also been reported to induce broader changes on the gut immune microenvironment, such as marked decrease of intraepithelial TCRγδ^+^ and CD8^+^ lymphocytes and reduced expression of the activating receptor NKG2D (96). Quite unexpectedly, there were also significant changes in gut microbial composition with significantly suppressed cecal *Clostridium* cluster *IV*, altered cecal *Firmicutes*/*Bacteroidetes* ratio, and reduction in ileal *Lactobacillus* spp. This was also associated with reduced body weight, and decreased size of adipose tissue and adipokine expression (96). The latter raise the possibility that TL1A may affect microbiota-related metabolic pathways that regulate adipose tissue development. Collectively, the above data support an important role for the TL1A/DR3 system in the maintenance of mucosal homeostasis and a significant contribution, when unrestrained, to late IBD-related immunopathology.

Mechanistic evidence for the implication of TL1A/DR3 signaling in the effector pathways that mediate chronic inflammation was recently presented by Li et al. (97) who tested the effect of DR3 stimulation of DR3 deletion in the ileitis-prone SAMP1/YitFc mouse model of CD. They, first, showed that administration of an agonistic antibody against DR3 (4C12) prior to disease development markedly worsened the severity of ileitis in SAMP mice. The immunological effects of DR3 stimulation included overproduction of T_H_1 and T_H_2 cytokines, expansion of dysfunctional CD25^−^FoxP3^+^ and ILC1 cells, and concomitant reduction of CD25^+^FoxP3^+^ and ILC3 cells. By comparison, genetic deletion of DR3 effectively reversed the inflammatory phenotype in SAMP mice. This was associated with selective expansion of CD25^+^FoxP3^+^ over CD25^−^FoxP3^+^ cells and upregulation of IL-10. These data demonstrate a central, multicellular modulation of adaptive immunity by DR3, via the regulation of the relative abundance of T_regs_, T effectors, and ILCs, which, subsequently, dictates the progression of CD-like ileitis in SAMP mice. More recently, Castellanos et al., have shown that TL1A induced ILC3 expression of OX40L in MHCII^+^ ILC3s that supports pathogenic T cell responses in the T-cell depended colitis transfer model. ILC3-spesific deletion of DR3 protected mice from the development of colitis (38). It follows that modification of DR3 signaling holds promise toward being an effective means for restoring the immunological balance between protective and inflammatory lymphocytes at the intestinal mucosa.

### TL1A/DR3 as Mediators of Fibrosis

Fibrosis refers to the process of excessive accumulation of extracellular matrix due to increased connective tissue assembly and ineffective matrix remodeling. Mostly, but not always, it represents the end result of repeated cycles of tissue inflammation, ulceration, and repair that ultimately lead to scarring and decline in organ function (98). Despite the wealth of knowledge of inflammatory pathways that have resulted in largely effective anti-inflammatory biologic treatment for various autoimmune diseases, including IBD, the fibrogenetic cascades that result to tissue scarring remain relatively understudied and effective therapies to prevent or, more importantly, reverse fibrotic processes are currently lacking (99).

TL1A amplifies multiple immunological pathways that, when sustained, could be associated with the development of fibrosis. Interleukin 17A favors the development of fibrosis in experimental models of lung and skin fibrosis and has been found to be overexpressed in intestinal strictures of CD patients (100, 101). IL-13 has been implicated in murine experimental intestinal fibrosis, acting mostly through TGFβ, and also is upregulated in strictures of patients with CD (102, 103). Finally, TL1A-induced expansion of Treg populations with altered function in the periphery may theoretically promote local saturation with IL-13 and, most importantly, TGFβ, which is the key regulator of pro-fibrotic pathways and a major activator of mesenchymal cells (104). Interestingly, elevated TL1A and DR3 expression has been found in the SAMP1/YitFc model of murine ileitis that is phenotypically associated with the development of overt intestinal strictures (35).

TL1A transgenic mice develop IL-13-dependent inflammation of the small bowel (57). Phenotypically, TL1A-tg mice are characterized by small bowel wall thickening, especially at the terminal ileum, which is the usual site of stricture development in human CD. Pathology was characterized by enhanced infiltration of the lamina propria with inflammatory cells, increase of the numbers and size of goblet and Paneth cells and hypertrophy of the muscularis propria (56, 57). These changes were accompanied by increased expression of IL-5, IL-13, and IL-17 by intestinal tissue and mesenteric lymph nodes.

Another set of studies by a different group on transgenic mice that constitutively expressed TL1A on the lymphoid or myeloid compartment have demonstrated a similar phenotype characterized by ileitis and Paneth cell hyperplasia (69). However, they further describe increased accumulation of collagen in intestinal tissue. Examination of the same transgenic mice under colitogenic conditions, in the context of the DSS and the adoptive T cell transfer model, revealed the development of overt intestinal strictures at the small and the proximal large bowel (105). The fibrotic phenotype was associated with increased local expression of TGFβ and IGF in colitic mice. Interestingly, constitutive production of TL1A was associated with relative expansion of CD4^+^IL17^+^ effector T cells in the mesenteric lymph nodes in the DSS by not in the adoptive T cell transfer model. This may indicate that diverse, TL1A-mediated profibrotic immunological pathways may dominate depending on the colitogenic conditions.

Further studies by the same group demonstrated that treatment with antibodies against TL1A was capable to reduce inflammation and to reverse fibrosis in both DSS and adoptive transfer model even when treatment was administrated late in the course of disease, that is after inflammation and fibrosis had been established (106). Anti-TL1A treatment decreased expression of pro-fibrotic molecules such as IGF1, CTGF, and TIMP1 in the inflamed intestinal tissue. Furthermore, mucosal expression of DR3 was associated with fibrotic changes in the bowel wall and DR3^−/−^ mice exhibited reduced numbers of intestinal fibroblasts and myofibroblasts. Intestinal myofibroblasts responded to TL1A with increased expression of Col1a2 and IL-31Ra, a myofibroblast activation marker. Finally, neutralization of TL1A reduced expression of a-SMA and vimentin, activation markers of colonic fibroblasts, and expression of TGFβ1 and Smad3 in the colonic tissue of adoptively T cell transferred colitic mice (107). However, only a subset (25%) of intestinal myofibroblasts expressed DR3, and the relative contribution of the TL1A/DR3 system on fibrosis independent of its anti-inflammatory effects was not explored. Despite this, these studies demonstrated for the first time that an anti-inflammatory therapy, in this case TL1A neutralization, can not only prevent but also potentially reverse established intestinal fibrosis. This is especially important for human CD which often has a long indolent course resulting in both inflammatory and fibrostenotic segments of the small bowel on initial patient presentation and disease diagnosis.

A recent study by Jacob et al., suggests that the pro-fibrotic effects of TL1A on bowel mucosa may depend on the composition of the intestinal microflora (108). TL1A-Tg mice raised under germ-free condition were protected from spontaneous ileitis and cecal collagen deposition. A direct effect of the host microflora was demonstrated on colonic fibroblasts exhibiting enhanced migration/proliferation and collagen production when derived from specific pathogen free as opposed to germ free littermates, and on wild type fibroblasts exposed to specific pathogen free microflora. Interestingly, gnotobiotic TL1A-Tg mice colonized with human gut microflora were protected from both ileitis and cecal fibrosis. Through 16S rRNA sequencing characterization of ileal and cecal microbiome the authors were able to depict possible bacterial genera and species that differentially promote fibrosis in the respective localizations in the context of TL1A overexpression (108).

In human fibrotic conditions, including IBD, the immunological and profibrotic cascades driven by TL1A remain underexplored. The potential implication of TL1A in such pathways was recently highlighted by the report of TL1A expression by human intestinal myofibroblasts that were isolated from IBD patients (109). TL1A expression by intestinal myofibroblasts was up-regulated by pro-inflammatory cytokines (IFN-γ, TNF-α, IL-1α) or supernatants of intestinal tissue cultures from IBD patients (109). The same pro-inflammatory cytokines induced expression of DR3 and DcR3 on cultured epithelial cells, whereas, supernatants from cultures of stimulated epithelial cells were capable to induce upregulation of TL1A in intestinal myofibroblasts. Similar findings have been reported for lung fibrosis and indicate the existence of additional TL1A-dependent possibly pro-fibrotic cascades mediated by epithelial/stromal cell interactions (110). These results point to the existence of a mucosal amplification loop that is initiated by the local pro-inflammatory milieu and then perpetuates itself through reciprocal stimulation of epithelial cell and intestinal myofibroblasts. Nevertheless, whether such interaction leads to increased collagen accumulation and fibrosis remains to be shown. An approach toward the elucidation of the relative contribution of TL1A/DR3 signaling in intestinal fibrosis independently from its role in inflammatory cascades would be the generation and study of conditional DR3 and TL1A knockout mice with tissue specific TL1A and DR3 deficiency on intestinal fibroblasts or epithelial cells (106).

## TL1A/DR3 in the Crossroads of Systemic Inflammation: Associations With Extraintestinal Inflammation

The TL1A/DR3 pathway is considered one of the common denominators in various pathologies associated with inflammation in different tissues that develops through aberrant immune responses. Genetic studies support its implication in autoimmune diseases and mycobacterial infections. Case-control and GWA studies have identified *Tnfsf15* gene variants, such as rs6478108 alleles, that associate with increased susceptibility to psoriasis and psoriatic arthritis in populations of European descent (111, 112). Furthermore, alleles rs6478108 (T) and rs4979462 (T), have been found to increase susceptibility to both Primary Billiary Cholangitis (PBC) and CD, while protecting from leprosy (6, 113). Moreover, the rs6478108 (G) allele has been associated with increased risk for CD and the pathological inflammatory host response in leprosy known as Type 1 reaction (114, 115).

Further genetic evidence links the TL1A/DR3 pathway to major extraintestinal manifestations of IBD. Indeed, genetic variation in the *Tnfsf15* gene has been associated with increased susceptibility in spondyloarthropathies, ankylosing spondylitis, and anterior uveitis (116–118).

Similarly to intestinal inflammation, increased TL1A was found in the serum of patients with rheumatoid arthritis and ankylosing spondylitis and also correlated with disease activity in both conditions (41, 119). More importantly, TL1A levels are decreased following treatment with anti-TNF. Local levels of TL1A have also been found increased in rheumatoid arthritis with mononuclear phagocytes being the major source of TL1A in the synovial tissue and synovial fluid of rheumatoid factor positive patients (40). In murine models of antigen induced arthritis and collagen induced arthritis TL1A administration promoted osteclastogenesis and exacerbated disease, whereas anti-TL1A ameliorated pathology (71). TL1A effects were found to be DR3-dependent as DR3^−/−^ mice were protected from cartilage depletion and joint destruction. This was partly attributed to decreased production of CXCL1 in the joints of DR3^−/−^ mice and the relative reduction of infiltrating neutrophil numbers which correlated with decreased local levels of MMP-9 (120).

Psoriasis is a common concomitant immune-mediated disorder in patients with IBD. Psoriasis and IBD share pathogenetic pathways such as the IL-23/IL-17 pathway, have common genetic risk alleles and are associated with abnormalities in intestinal and skin microflora and both respond to anti-TNF and anti-p40 treatment (121). There is evidence that TL1A/DR3 may represent an additional pro-inflammatory pathway that is shared between the two diseases. TL1A and DR3 have been found to be increased in psoriatic skin lesions and specifically in macrophages and keratinocytes (122). TL1A seems to synergize with IL-23 to stimulate PMBCs from patients with psoriasis to increase production of IL-17 (123). Uveitis is another extraintestinal manifestation of IBD that has been associated with the TL1A/DR3 pathway. Specifically, experimental murine autoimmune uveoretinitis was dependent on DR3 as DR3^−/−^ mice are protected from disease development (124). Although a direct association of TL1A/DR3 with the extraintestinal manifestations of IBD has not been established yet, there is indirect evidence that such an association may exist making TL1A/DR3 a possible common denominator of the gut-skin-joint-eye autoimmune inflammation axis.

## Concluding Remarks

Since its initial description, TL1A has arisen as an important mucosal factor that is implicated in homeostasis and inflammation through its association with DR3. In the light of translational medicine, it is important that this role of TL1A/DR3 is supported by converging lines of evidence. In particular, polymorphisms in *Tnfsf15* significantly affect susceptibility to IBD and may be associated with altered function of the respective protein. In addition, there is significant upregulation and abundant expression of TL1A and DR3 in inflammatory conditions that affect the intestines, mainly IBD. So far, functional properties of the TL1A/DR3 system involve several immunological pathways that are considered important in the pathogenesis of IBD. Finally, the proof of concept for the therapeutic application of TL1A/DR3 modification has been fulfilled in animal models of intestinal inflammation. In all, it could be said that the system of TL1A/DR3 may represent a desirable therapeutic target for a subset of IBD patients.

Nevertheless, caution is also required as important questions remain unanswered still. One area of concern is the potential effect on Tregs. As TL1A/DR3 have positive effects on Treg function, their neutralization may compromise this population and jeopardize its important anti-inflammatory function in intestinal immunity. A second question is whether blockade of TL1A or DR3 should be the preferable approach in clinical practice. This is of importance as recent evidence from animal models of inflammation reported similar but not identical effects of genetic or immunologic deletion of the two molecules. Finally, antibodies against DR3 have shown agonistic effects which depend on the target population. All these parameters should be taken into account for the design of clinical trials that aim to disrupt TL1A/DR3 signaling.

## Author Contributions

VV, GK, and GB all contributed to the development of the concept of the review, performed the literature review and analyzed existing data, and wrote and edited the manuscript.

### Conflict of Interest Statement

The authors declare that the research was conducted in the absence of any commercial or financial relationships that could be construed as a potential conflict of interest.
